# Adult Granulosa Cell Tumors of the Ovary: A Retrospective Study of 36 FIGO Stage I Cases with Emphasis on Prognostic Pathohistological Features

**DOI:** 10.1155/2018/9148124

**Published:** 2018-08-16

**Authors:** Emina Babarović, Ivan Franin, Marko Klarić, Ani Mihaljević Ferrari, Ružica Karnjuš-Begonja, Senija Eminović, Damjana Verša Ostojić, Danijela Vrdoljak-Mozetič

**Affiliations:** ^1^Department of Pathology, School of Medicine, Rijeka University Hospital Centre, School of Medicine, University of Rijeka, Braće Branchetta 20, Rijeka, Croatia; ^2^School of Medicine University of Rijeka, Braće Branchetta 20, Rijeka, Croatia; ^3^Clinical Department of Obstetrics and Gynecology, Rijeka University Hospital Centre, School of Medicine, University of Rijeka, Krešimirova 42, Rijeka, Croatia; ^4^Clinical Department of Radiotherapy and Oncology, Rijeka University Hospital Centre, School of Medicine, University of Rijeka, Krešimirova 42, Rijeka, Croatia; ^5^Department of Cytology, Rijeka University Hospital Centre, Krešimirova 42, Rijeka, Croatia

## Abstract

**Objective:**

Adult granulosa cell tumors (AGCTs) represent 2%–5% of all ovarian malignancies. The aim of this study was to analyze clinical and pathohistological parameters and their impact on recurrence, overall, and disease-free survival in FIGO stage I AGCT patients.

**Methods:**

The tumor specimens analyzed in this retrospective study were obtained from a total of 36 patients with diagnosis of ovarian AGCT surgically treated at the Department of Gynecology, Rijeka University Hospital Centre, between 1994 and 2012. Clinical, pathological, and follow-up data were collected.

**Results:**

The mean age at diagnosis was 54.5 years with a range of 24–84. The majority of the patients, 30 (83%), were in FIGO stage IA, 3 (8%) in stage IC1, 1 (3%) in stage IC2, and 2 (6%) in stage IC3. During follow-up period (median 117.5 months, range 26–276), recurrence occurred in 4 patients (12%) with 2 deaths of the disease recorded. In univariate analysis, the 5-year survival rates were significantly shorter in patients with FIGO substage IC (*p* = 0.019), with positive LVSI (*p* = 0.022), with presence of necrosis (*p* = 0.040), and with hemorrhage (*p* = 0.017). In univariate analysis, the 5-year disease-free survival rates were significantly shorter in patients treated with fertility surgery (*p* = 0.004), with diffuse growth pattern (*p* = 0.012), with moderate and severe nuclear atypia (*p* = 0.032), and with presence of hemorrhage (*p* = 0.022). FIGO substage IC proved to be independent predictor for recurrence (OR = 16.87, *p* = 0.015, and OR = 23.49, *p* = 0.023, resp.) and disease-free survival (*p* = 0.0002; HR 20.84, *p* = 0.02) at the uni- and multivariate analyses.

**Conclusions:**

FIGO substage IC is predictive of recurrence and disease-free survival in patients with early-stage AGCTs. LVSI, presence of necrosis and hemorrhage, diffuse growth pattern, and nuclear atypia in AGCTs seem to be associated with overall and disease-free survival, so these pathological features should be taken into consideration when managing patients with AGCT.

## 1. Introduction

Adult granulosa cell tumors (AGCTs) are an uncommon type of ovarian neoplasm that represent approximately 2%–5% of all ovarian malignancies [1, 2]. AGCTs occur more often in middle-aged and postmenopausal women, with a peak incidence between 50 and 55 years of age [3]. Clinically, they are the most common ovarian tumors that secrete hormones, primarily estrogen, so they can cause endometrial alterations like hyperplasia and endometrial cancer and patients are often presented with irregular vaginal bleeding. These malignancies are often low grade and have low clinical stage at presentation, but with an awkward biological potential for late recurrence [4–6]. The majority of women with AGCTs are diagnosed at stage I disease, and overall the prognosis for these women is quite good, with several authors reporting a 10-year survival rate of more than 80% [1, 3]. Nevertheless, the main clinical characteristic of these tumors is a tendency for late recurrence. Overall, recurrence can be expected in ~20% of patients with early-stage AGCTs [2, 4, 6]. The average time to recurrence is 5 years after surgical treatment of the primary tumor, but numerous cases of recurrence have been reported in the literature even after 20 to 30 years of initial diagnosis [7].

A recent study by Shah et al. demonstrated that a single somatic point mutation in the transcription factor FOXL2 is present in tumor samples from patients with AGCTs [8]. The studies that followed have proven that FOXL2 mutation was present in virtually all AGCTs but was negative in other tumor types [9, 10]. Based on these evidences, this mutation is now considered as a defining feature of AGCTs and is a powerful diagnostic tool in the differential diagnosis of these tumors [11–14]. Furthermore, there are also indications that FOXL2 is a driver of AGCT pathogenesis and could be a potential target for future therapeutics [15].

Surgery is the mainstream of initial treatment aiming at achieving the histological diagnosis and appropriate staging [5, 6, 16–20]. Standard surgical treatment of ovarian AGCTs has changed over the years. Namely, recent published series suggested a lack of lymph node involvement and recommended abandonment of the lymph node dissection as part of the primary surgical staging of these tumors [16, 19, 20]. Tumor stage is the most important prognostic factor in AGCTs [3, 6, 14]. Numerous studies have attempted to define other prognostic factors like patient's age, tumor size, grade of differentiation, mitotic activity, and lymphovascular space invasion [2–5, 16–18, 20–25]. However, their role as prognostic factors has not been clearly determined, because of the rarity of these tumors and the need for very long follow-up period. Determination of reliable and more accurate histopathologic features and biological markers of ovarian AGCTs is required to aid gynecologic oncologists for stratification of patients into risk groups and to predict which patient has higher risk for development of recurrence to enable the additional refinement of adjuvant treatment recommendations.

Taking into account the abovementioned and existing contradictory clinical and pathohistological prognostic data, the aim of our study was to analyze the clinical and pathohistological parameters and their impact on recurrence, overall, and disease-free survival in a homogeneous group of patients with adult granulosa cell tumors in clinical stage I disease.

## 2. Material and Methods

The tumor specimens analyzed in this retrospective study were obtained from a total of 36 patients with diagnosis of AGCTs of the ovary surgically treated at the Department of Gynecology, Rijeka University Hospital Centre, between 1994 and 2012. Pathology databases were used to identify patients. All available computerized and paper medical records, including inpatient and outpatient visits at the institution, were reviewed. Data was collected on patient characteristics, clinical findings, and all treatment received for primary or recurrent disease. The study included all consecutive patients for which all clinical data and adequate tumor specimens were available. We included only the patients operated on till 2012 to ensure the follow-up of a minimum of 5 years. All patients were staged according to the new International Federation of Gynecology and Obstetrics (FIGO) classification (2014) for ovarian cancer. Only patients with FIGO stage I disease were included in this retrospective study. Exclusion criteria included cases with concomitant diagnosis of another malignancy that was not an adult granulosa cell tumor or an endometrial carcinoma. Clinical features of the patients are presented in Table 1.

All archived histopathology slides for hematoxylin and eosin (H&E) stain, of the cases that met the inclusion criteria, were pulled and examined by two expert gynecologic pathologists (SE and EB). The pathologists who evaluated the slides in this cohort were completely blinded to the clinical information. Clinical and pathological variables included patient age, surgical procedure, and final pathological analysis with gross appearance of the tumors and detailed microscopic features. More specifically, we analyzed presence of necrosis and hemorrhage in tumor tissue, the pattern of granulosa cell growth, and the number of patterns admixed in one tumor. The predominant growth pattern was evaluated for each tumor and for the purpose of statistical analysis; the tumors were divided into two groups: those with predominantly diffuse growth pattern versus tumors with other growth patterns. Nuclear atypia was also evaluated, and tumors were divided into two groups: tumors with no or with mild nuclear atypia versus tumors with moderate and severe nuclear atypia. The mitotic index was expressed in terms of the number of unequivocal mitotic figures per 10 high-power fields (HPF). The slides were screened at low magnification for finding the area with the highest density of mitotic figures, and then mitotic counts were performed at HPF in three sets of ten randomly chosen contiguous fields. The mitotic index is expressed as the average number of mitotic figures per 10 HPF. According to other studies in the literature, we set the cutoff at 4 mitotic figures per 10 HPF to divide tumors into low and high category [23–25]. Lymphovascular space invasion (LVSI) was diagnosed only when viable tumor nests were observed within endothelial-lined spaces with or without intraluminal red cells or lymphocytes. Areas of potential artifact, clefts, or tumor cell contamination (torn tissue, free tumor fragments along the edge of the tissue) were all ruled out. No attempts were made to differentiate between lymphatic and vascular vessels because of the difficulty and lack of reproducibility that is associated with routine light microscopy. We scored LVSI in a dichotomized fashion: positive (present) or negative (absent). The interobserver concordance was excellent; in individual cases of interobserver variation, a third reviewer evaluated the discordances. This group of tumors is composed of various combinations of granulosa cells, theca cells, and fibroblasts. The presence of at least 30% of fibroma/thecoma component in tumor tissue was used to put cases in AGCTs with prominent fibrothecomatous component.

The study was approved by the Clinical Medical Hospital Rijeka Ethics Committee and Faculty of Medicine University of Rijeka Ethics Committee for biomedical research.

### 2.1. Statistical Analysis

Statistical analysis was performed using MedCalc for Windows, version 18 (MedCalc Statistical Software bvba, Ostend, Belgium). The classical descriptive methods were used as well as the Fisher exact test and *χ*^2^-test for the comparison of proportions. Mann–Whitney test was used to compare the medians. Correlation was studied using Kendall tau rank correlation. The method of logistic regression was used in order to compute the odds ratio (OR) for predictors of recurrence in a univariate and multivariate manner. The association of analyzed parameters with patient overall and disease-free survival was evaluated using the Kaplan-Meier method, and differences between survival curves were tested for statistical significance using the log-rank test. Parameters with prognostic significance were included in multivariate Cox regression model. In all tests, the level of significance was set at *p* < 0.05.

## 3. Results

### 3.1. Clinicopathological Features

The median age at diagnosis was 54.5 (range 24–84) years. Thirty patients (83%) were in FIGO stage IA and 6 (17%) in stage IC. None of the patients were in FIGO stage IB, so all our patient had unilateral disease with slight predominance of the right ovary (19 patients, 53%). Besides that, none of the patients in our cohort presented with tumor rupture and haematoperitoneum. The majority of patients were treated by total abdominal hysterectomy with bilateral salpingo-oophorectomy (TAH + BSO) with or without complete staging. Complete staging included peritoneal washings, peritoneal biopsies, appendectomy, and omental sampling with or without pelvic and paraaortic lymph nodal sampling. Lymphadenectomy was performed in 6 patients (17%), pelvic with paraaortic lymphadenectomy in 4 patients, and only pelvic lymphadenectomy in 2 patients. There were inconsistency in staging surgery modalities due to wide treatment era and the unpredictable diagnosis of AGCT. Pelvic and/or paraaortic lymphadenectomy were optional procedures, according to the surgeon's experience and the intraoperative findings, so systematic complete pelvic and paraaortic lymphadenectomy during primary surgery was done in 1 patient with concomitant endometrial carcinoma, and the remaining lymphadenectomies were done in patients with larger tumors for whom we had an intraoperative diagnosis at the frozen section. Paraaortic lymphadenectomy was not technically feasible in 2 patients so in those cases only pelvic lymphadenectomy was performed. The median number of received lymph nodes was 20 with a range of 10–36, all of which were histologically negative. Seven patients were treated with unilateral salpingo-oophorectomy with staging. These were mostly younger patients with a view to maintaining fertility or in patients who had a previously operated uterus and other adnexa due to benign pathology. Seventeen patients (51%) had endometrial alterations, that is, hyperplasia without atypia was present in 13 patients (39%), complex hyperplasia with atypia was present in 3 patients (9%), and 1 patient (3%) had well differentiated endometrioid adenocarcinoma of the endometrium, staged according to FIGO classification IB. There were only 3 patients without a specimen of endometrium for evaluation. Four patients were lost to follow-up. Postoperative adjuvant chemotherapy with 4 cycles of BEP protocol (bleomycin, etoposide, and cisplatin) was administered in 3 patients who were in stage IC disease, and 2 of those patients developed recurrence. In 1 patient out of the remaining 3 with stage IC disease for which adjuvant chemotherapy was not administered, recurrent disease occurred. The patients were examined every 3 months for the first 2 years, every 6 months for the next 3 years, and yearly thereafter. During the follow-up period, recurrence occurred in 4 patients and 2 of them died of the disease. Among the 4 patients with recurrent disease, only 1 had isolated pelvic recurrence and the other 3 patients suffered multiple recurrences: 2, 3, and 5, respectively. Clinical, pathological, and treatment details of patients with recurrent disease are presented in Table 2.

The median months to occurrence of first recurrence were 40 with a range of 6–120 months. The date of recurrence was determined by clinical examination, imaging studies, and CA 125 levels. In addition to that, recurrent diseases of all our patients were treated surgically so histological slides of recurrent tumor were also available to confirm the recurrence. Overall survival was measured from the day of diagnosis until the last follow-up visit to the outpatient clinic (censored patients) or death. Eight of the 32 patients for whom complete follow-up was available had died, and 6 of them died due to a cause other than AGCT, so they were also censored. The median follow-up time was 117.5 months (range 26–276 months). Disease-free survival was defined as the time interval from the date of diagnosis to the date of documented first recurrence of disease. If there was no recurrence, disease-free survival was determined as the date of last follow-up.

### 3.2. Association of Clinicopathological Features with Disease Recurrence

On gross examination, the median tumor size was 6.6 cm with a range of 0.7–30 cm. Eight patients (23%) had tumor size 10 cm or greater. 21 patients (58%) presented with an entirely solid tumor mass, yellow to gray on the cut surface; 6 patients (17%) had an exclusively cystic tumor; and 9 (25%) presented with a combination, cystic, and solid tumor mass. Necrosis was present in the tumor tissue of 8 (22%) patients and abundant hemorrhage in 10 tumors (28%). No differences in the tumor size (*p* = 0.083), gross appearance (*p* = 0.886), and presence of necrosis (*p* = 1.00) between patients with and without recurrent disease were observed. The observed higher frequency of hemorrhage in the tumor tissue of the patients with recurrent disease did not reach statistical significance (*p* = 0.056). Besides that, a weak but statistically significant positive correlation between hemorrhage in the tumor tissue and LVSI was observed (*r* = 0.212, *p* = 0.05).

The majority of tumors, 30 (83%), had at least two (28%) or three and more (55%) multiple, admixed growth patterns of granulosa cells. The predominant growth pattern in our cohort was distributed as follows: 17 (47%) samples with diffuse growth pattern, 8 (22%) microfollicular, 5 (14%) insular, 4 (11%) corded or trabecular, and 2 (6%) macrofollicular. When we compared the growth pattern with the recurrence of the disease, a significantly higher frequency of diffuse growth pattern was observed in patients who developed recurrent disease; in fact, in our cohort all patients with recurrence had predominantly diffuse growth pattern in primary tumor (*p* = 0.004). Surprisingly, there was no correlation between the tumor size and predominantly diffuse growth pattern; moreover, tumors larger than 10 cm did not have a higher frequency of multiple growth patterns (*p* = 0.913) or higher frequency of predominantly diffuse growth (*p* = 0.706). There was no differences in the number of growth patterns (*p* = 0.118), grade of cytological atypia (*p* = 0.113), mitotic index (*p* = 1.00), and presence of prominent fibrothecomatous component (*p* = 0.609) between patients with and without recurrent disease. We detected positive LVSI in 11 tumor specimens (31%) but the observed higher frequency of LVSI in tumor specimens of patients with recurrent disease did not reach statistical significance (*p* = 0.075). The differences of clinicopathological features between patients with and without recurrent disease are summarized in Table 3.

Univariate analysis revealed a statistical significant association with disease recurrence of FIGO substage IC (OR 29.0; CI 2.25–373.79; *p* = 0.009) and fertility surgery (OR 21.0; CI 1.73–254.29; *p* = 0.017). Patient age, tumor size, necrosis, hemorrhage, diffuse growth pattern, grade of cytological atypia, mitotic index, and LVSI were not statistically significant predictors of disease recurrence. On multivariate analysis, only the FIGO substage retained significance (OR19.34; CI 1.10–339.18; *p* = 0.043; AUC 0.941) and was an important prognostic factor for disease recurrence.

### 3.3. Survival Analysis

The association of analyzed parameters with overall patient survival during follow-up is shown in Table 4. In the univariate analysis, the 5-year survival rates were significantly shorter in patients with FIGO substage IC (*p* = 0.019), with positive LVSI (*p* = 0.022), with presence of necrosis (*p* = 0.040), and with hemorrhage (*p* = 0.017) in the tumor tissue. On the multivariate model, the level of significance was not retained for neither of the parameters.

The association of analyzed parameters with disease-free survival of patients is shown in Table 5. In the univariate analysis, the 5-year disease-free survival rates were significantly shorter in patients with FIGO substage IC (*p* = 0.0002), treated with unilateral salpingo-oophorectomy with staging (*p* = 0.004), with predominant diffuse growth pattern of granulosa cells (*p* = 0.012), with moderate or severe nuclear atypia of tumor cells (*p* = 0.032), and with presence of hemorrhage in the tumor tissue (*p* = 0.022). Observed shorter 5-year disease-free survival rates in patients with positive LVSI in the tumor tissue only show a strong trend (*p* = 0.058) so we suspect that it would also become statistically significant in a larger patient cohort. On the multivariate analysis, only the FIGO substage retained significance (HR 20.84; CI 1.48–292.95; *p* = 0.02) and was an important independent prognostic factor for disease-free survival of patients.

## 4. Discussion

Practically, 80–90% of patients with AGCTs are presented in FIGO stage I disease [1]. The overall prognosis for these patients is very good [1, 3, 6]. Surgery is the standard initial treatment modality for all patients with AGCTs, and postoperative adjuvant chemotherapy of AGCT has changed and evolved over the past decades [19, 26–28]. Despite advances in therapy and seemingly favorable clinical behavior, some patients diagnosed with early-stage AGCT suffer from recurrence and disease-related mortality [16, 25, 29, 30]. As with other rare tumors, detailed information on the natural history and optimal management of patients with AGCTs is limited. In addition to that, its slow progression and required long-time period of follow-up make it even more difficult to understand the biological behavior of the disease. So, the optimal management of patients has not yet been satisfactorily established, and more specific determination of the high-risk pathohistological features of AGCTs are required to aid gynecologic oncologist in the stratification of patients into risk groups and planning of adjuvant therapy.

In this study, we wanted to analyze the clinical and detailed pathohistological prognostic parameters in a homogeneous model of early-staged AGCT patients with long-term follow-up.

Among our patients, 17 presented with endometrial alterations, concomitant endometrial carcinoma, or hyperplasia, which underlies the need for biopsy of the endometrium during conservative procedures in order to exclude a concomitant neoplasia. This observation is consistent with other studies conducted [31].

AGCT can affect younger patients and fertility preservation is an important issue. However, the role of fertility surgery remains unclear. Some studies have found that fertility surgery was associated with high recurrence and lower survival rates [2, 32], while others found no difference in recurrence rates between fertility and radical surgery in patients with stage I disease [33]. In our study, patients who underwent fertility surgery had more frequently recurrent disease, and in univariate analysis fertility surgery was associated with disease recurrence and shorter disease-free survival but it did not retain significance in a multivariate manner. Nevertheless, close monitoring of these patients can be recommended with hysterectomy and salpingo-oophorectomy after completion of family planning.

Given the negative lymph nodes in our patients with lymphadenectomy and low rate of involvement of lymph nodes at recurrence (only 1 patient in our cohort with positive retroperitoneal lymph nodes, but only in the third recorded recurrence), we are in agreement with previously conducted studies which implicated that staging of AGCTs should not be extended to lymphadenectomy [16, 18–20, 29, 34]. Opposite to that, Bryk et al. in their study of 164 molecularly defined AGCTs have suggested that patients in FIGO substage IC might benefit from complete staging including lymphadenectomy and adjuvant chemotherapy, although these results were evident only in univariate analysis [35].

The role of adjuvant chemotherapy for FIGO substage IC patients is hugely debatable, and for that reason only 3 out of 6 our patients received postoperative adjuvant chemotherapy with 4 cycles of BEP protocol. Two of our patients that received adjuvant therapy developed recurrence, but it was impossible to draw any meaningful conclusions regarding the role of adjuvant chemotherapy on such a small sample. Current National Comprehensive Cancer Network (NCCN) guidelines recommend adjuvant chemotherapy for advanced-stage patients and stage I disease with high risk factors, but clear definition of what constitutes a high risk factor remains controversial. Two recent studies by Mangili et al. [36] and Wang et al. [37] that analyzed AGCT patients in FIGO substage IC failed to demonstrate the benefit of adjuvant chemotherapy. The results of their studies have shown that adjuvant chemotherapy was not associated with improved DFS and did not protect patients against recurrence of the disease. The largest retrospective study thus far that included a total of 739 patients with 570 stage I and 139 substage IC patients also failed to detect an association between adjuvant chemotherapy and improved DFS in any of the subgroups or in the entire study cohort [38]. The potential serious toxicity of administered BEP chemotherapy such as myelosuppression and pulmonary disorders with a lack of obvious benefit suggests that new studies are needed to address the question of decision to administer chemotherapy in early-stage AGCT [39]. FOXL2 mutation was associated with increased CYP17 expression, and inhibition of this enzyme may be achieved by novel agents such as abiraterone and ketoconazole [40]. This new potential target for future therapeutics may prove to be more useful and less toxic and maybe could be considered in the adjuvant setting.

Studies have proposed several risk factors for recurrence after surgery for AGCT including advanced age, large tumor size, tumor rupture, LVSI, degree of cytological atypia, and high mitotic index [1, 3, 18, 20, 33, 41–46]. The effect of the abovementioned predictors is controversial with different results of studies in the literature. However, we need to know that conducted studies have important limitations mainly due to heterogeneity of the population, small number of patients included, and some of them with not long enough follow-up period. We have to admit that our study, as well, has several limitations, including its retrospective nature and small sample size with small number of incidences (recurrences and deaths). But the rarity of AGCT makes it hard to carry out well-designed studies. Advantages of our study are focusing and analyzing a homogeneous group of patients with stage I disease minimizing the influence and biases of other potential factors like residual disease, higher stage, and different modalities of adjuvant treatment. An additional strength of our study is long-term follow-up period with a median of 9.8 years, which is significantly longer compared to some other studies [4, 5, 23, 24, 44].

Age has not been consistently correlated with survival or recurrence of women with AGCTs [2, 5, 18, 23, 25, 31, 46]. Zhang et al. [43] found that women younger than 50 years had a 10% survival advantage in a cohort of 376 women. Additionally, Ayhan et al. [23] reported that women younger than 60 years survived longer and had less mortality. Contrary to these findings, Bryk et al. [35] have shown that premenopausal patients have higher rate of relapses and in their study age less than 40 years was associated with increased rate of relapses. Our study is in accordance with other conducted studies, and we did not demonstrate difference in age between groups of patients regarding recurrence, and age was not a significant predictor of overall or disease-free survival [2, 5, 18, 25].

Thrall et al. [18] in their study demonstrated that tumor size was the most important predictor of death from disease, with no disease recurrences noted among women with primary tumors smaller than 7 cm. Tumor size has been noted to be an important prognostic factor in many reports [1–3, 5, 20, 25, 46]. In these series, tumors larger than 10 to 15 cm were associated with an increased risk of recurrence and death from disease. Conversely, other authors have suggested that tumor size is not an important prognostic factor after adjusting for other clinical factors such as stage, and some studies were unable to validate the prognostic significance of tumor size which is consistent with our results [4, 22, 23, 29, 44].

Disease stage has been demonstrated to be the most important predictor of survival to date [2, 3, 14–16, 23–25, 33, 43–47]. The results of our study demonstrated that FIGO substage IC (surgical spill, capsule rupture before surgery, tumor cells on ovarian surface, and positive peritoneal washings) is an important and independent prognostic factor for disease recurrence and recurrence-free survival in patients with early-stage AGCTs. So, as far as stage is concerned, our study is in accordance with others and confirms that even in early-stage patients, higher substage is one of the most important predictors for recurrence. Only one patient in our group with recurrent disease had FIGO stage IA disease, and the remaining three patients were in the IC substage. Our patient with FIGO stage IA disease had AGCT with predominantly diffuse pattern of growth and positive LVSI in the tumor tissue. She developed first recurrence 10 years after the diagnosis, then second after 14 years, and third after 15 years and unfortunately died of the disease 20 years after the diagnosis, so we believe that long-term follow-up is necessary in order to obtain reliable data regarding important pathohistological features of AGCTs. Most of the studies that evaluated classical pathohistological features of AGCTs are older, and today we are aware that these studies of granulosa cell tumors are suspects because they almost certainly included tumors that are not granulosa cell tumors; thus, drawing conclusions regarding the impact on survival and recurrence are not recommended. However, recent studies have shown that FOXL2 mutation is a defining feature of AGCTs so this is important for future studies, especially those including advanced stage of the disease, which should include only molecularly defined granulosa cell tumors [8–15, 35].

We observed more frequent diffuse pattern of growth in AGCTs of patients with recurrent disease. Namely, all our patients with recurrence had predominantly diffuse growth pattern of neoplastic granulosa cells. Furthermore, diffuse growth pattern was associated with shorter disease-free survival. Our observations are not in accordance with a study by Fujimoto et al. [24]. In their study of 27 patients with AGCT, they have demonstrated that microscopic patterns of growth were not associated with disease-free survival.

LVSI can be not only a clinical prognostic parameter but also a sign of invasive and metastatic potential of granulosa cells. But, in literature there is an extremely wide range of reported incidence of LVSI in patients with AGCT. For example, Fox et al. [5] reported lymphatic invasion in only 4 out of 92 cases of granulosa cell tumors (4.3%); on the other hand, Fujimoto et al. [24] reported LVSI in 11 out of 27 cases of AGCT (40.7%), which is as a result closer to our incidence of 31% (11 out of 36 cases). This difference could be due to the use of different criteria for diagnosis of lymphatic or blood vessel invasion. We are not sure because some investigators did not describe their criteria. Fujimoto et al. [24] in their study used criteria of General Rules of the Japanese Research Society for Gastric Cancer for assessing LVSI, so they classified LVSI in 4 categories: none, minimal, moderate, and considerable. They had patients with AGCT at all stages of the disease (17 cases stage I, 4 stage II, 5 stage III, and 1 stage IV), so like expected their incidence of LVSI was somewhat higher than ours. Just as well, it was reasonable to rate LVSI in their patient group where it is expected to have higher incidence of invasion, but we believe that for our study group of early-stage AGCT patients, the best way to score LVSI was in a dichotomized fashion: positive or negative. Previous data have suggested that mitotic index can be a prognostic marker in granulosa cell tumors [4, 5, 24, 39], but this has not been confirmed in all studies and our results are in accordance with them [1]. Malmstrom et al. [4] demonstrated that patients in stage I disease have significantly shorter survival when having 10 or more mitotic figures per 10 HPF. In a more recent study by Fujimoto et al. [24], higher mitotic index and LVSI were independent prognostic factors of disease-free survival in patients with AGCT. Our results suggest that positive LVSI is associated with significantly shorter overall survival of patients; moreover, we observed higher frequency of positive LVSI in tumor specimens of patients with recurrent disease and shorter 5-year disease-free survival rates in those patients, but those results are at the level of strong statistical trend, so we suspect that it would also become statistically significant in a larger patient cohort. Besides that, an interesting result of our study is a positive correlation between hemorrhage in the tumor tissue and LVSI. This observation can be useful for clinical pathologists when encountering AGCT with abundant hemorrhage to examine more carefully lymphovascular spaces and maybe take a few more samples of tumor tissue for evaluation of LVSI.

Some granulosa cell tumors in their microenvironment have abundant theca cells and fibroblasts. We wanted to investigate whether such a tumor microenvironment contributes to the aggressiveness of the tumor. The results of our study did not support this hypothesis, and prominent fibrothecomatous component in the tumor tissue was not associated with prognosis in our patients.

## 5. Conclusions

The key finding of our study is that FIGO substage IC is an important and independent prognostic factor for disease recurrence and recurrence-free survival in patients with FIGO stage I AGCTs. We believe that additional studies of LVSI, presence of necrosis and hemorrhage, diffuse growth pattern, and nuclear atypia in early-stage AGCTs are required because these pathological features seem to be associated with overall and disease-free survival, so these should be taken into consideration when managing patients with AGCT.

## Figures and Tables

**Table 1:** Clinical features of the patients with early-stage adult granulosa cell tumors.

Clinical features (*N* = 36)	
*Age (years)*	
Median	54.5
Range	24–84
*FIGO substage*	*Cases (%)*
IA	30 (83%)
IB	0
IC1	3 (8%)
IC2	1 (3%)
IC3	2 (6%)
*Initial surgical procedure*	*Cases (%)*
TAH + BSO	16 (44%)
TAH + BSO + staging op	13 (36%)
USO (fertility surgery)	7 (20%)
*Lymphadenectomy*	*Cases (%)*
Not performed	30 (83%)
Performed	6 (17%)
*Adjuvant therapy*	*Cases (%)*
Surgery only	32 (91%)
Surgery + chemotherapy	3 (9%)
*Endometrial pathology*	*Cases (%)*
None	16 (49%)
Hyperplasia without atypia	13 (39%)
Hyperplasia with atypia	3 (9%)
Carcinoma	1 (3%)
*Recurrence*	*Cases (%)*
No	28 (88%)
Yes	4 (12%)
*Months to recurrence*	
Median (range)	40 (6–120)
*Follow-up (months)*	
Median (range)	117.5 (26–276)
*Died of disease (N (%))*	2 (6%)

Note: TAH + BSO: total abdominal hysterectomy with bilateral salpingo-oophorectomy; USO: unilateral salpingo-oophorectomy.

**Table 2:** Clinical, pathological, and treatment details of patients with recurrent disease.

Case	Age (years)	FIGO	Tumor size	Grade of atypia	Mitotic index	LVSI	Initial treatment	Months to first recurrence	Site of recurrence	Treatment of recurrence	Number of recurrences (months)	Status and overall survival
1	51	IA	7	1	3	Positive	USO	120	Peritoneum, liver, adrenal gland, retroperitoneal lymph nodes	Surgery + Ch + HT	3 (168, 180)	DOD, 240
2	61	IC1	30	2	10	Positive	TAH + BSO, Om, App, peritoneal biopsies	41	Retroperitoneal fibrous and fat tissue, peritoneum, pelvis, hernia sac	Surgery + Ch	5 (43, 48, 57, 62)	DOD, 66
3	36	IC1	5	2	8	Negative	USO	38	Pelvis, ovary, peritoneum, fallopian tube, omentum	Surgery + Ch	2 (38, 41)	NED, 132
4	60	IC1	3	2	2	Positive	USO (uterus and other adnexa were earlier removed due to leiomyoma)	6	Pelvis	Surgery + Ch	1	NED, 54

TAH + BSO: total abdominal hysterectomy with bilateral salpingo-oophorectomy; USO: unilateral salpingo-oophorectomy; Om: omentectomy; APP: appendectomy; Ch: chemotherapy; HT: hormonal therapy; DOD: died of the disease; NED: no evidence of disease.

**Table 3:** Clinicopathological characteristics in comparison to recurrence of early-stage adult granulosa cell tumors.

Features	All (*N* = 36)	Recurrent disease		*p* value
		No (*N* = 28)	Yes (*N* = 4)	
Age (years)				
≤55 y	20 (56%)	18 (56%)	2 (50%)	1.00^±^
>55 y	16 (44%)	14 (44%)	2 (50%)	
FIGO substage				
IA	30 (83%)	29 (91%)	1 (25%)	**0.01** ^±^
IC	6 (17%)	3 (9%)	3 (75%)	
Surgery				
Radical surgery	29 (81%)	28 (88%)	1 (25%)	**0.018** ^±^
Fertility surgery	7 (19%)	4 (12%)	3 (75%)	
Tumor size				
<10 cm	26 (77%)	23 (77%)	3 (75%)	1.00^±^
≥10 cm	8 (23%)	7 (23%)	1 (25%)	
Tumor gross appearance				
Solid	21 (58%)	19 (59%)	2 (50%)	0.886^¶^
Cystic	6 (17%)	5 (16)	1 (25%)	
Cystic-solid	9 (25%)	8 (25%)	1 (25%)	
Growth patterns of AGCT				
Only 1	6 (17%)	4 (13%)	2 (50%)	0.118^¶^
Mixed with 2 patterns	10 (28%)	10 (31%)	0	
Mixed with 3 and more	20 (55%)	18 (56%)	2 (50%)	
Dominant growth pattern AGCT				
Diffuse	17 (47%)	13 (41%)	4 (100%)	**0.004** ^±^
Others	19 (53%)	19 (59%)	0	
Grade of cytologic atypia				
1	16 (44%)	16 (50%)	0	0.113^±^
2 and 3	20 (56%)	16 (50%)	4 (100%)	
Mitotic index				
<4	19 (53%)	17 (53%)	2 (50%)	1.00^±^
≥4	17 (47%)	15 (47%)	2 (50%)	
LVSI				
Negative	25 (69%)	24 (75%)	1 (25%)	0.075^±^
Positive	11 (31%)	8 (25%)	3 (75%)	
Necrosis				
Absent	28 (78%)	25 (78%)	3 (75%)	1.00^±^
Present	8 (22%)	7 (22%)	1 (25%)	
Hemorrhage				
Absent	26 (72%)	25 (78%)	1 (25%)	0.056^±^
Present	10 (28%)	7 (22%)	3 (75%)	
Prominent fibrothecomatous component				
Absent	23 (64%)	21 (66%)	2 (50%)	0.609^±^
Present	13 (36%)	11 (34%)	2 (50%)	

^±^Fisher exact test; ^¶^chi-squared test; AGCT: adult granulosa cell tumor; LVSI: lymphovascular space invasion.

**Table 4:** Univariate analysis of overall survival in patients with early-stage adult granulosa cell tumors.

Features	*N*	Died of the disease	% 5-year survival	Log-rank test (*χ*^2^-test) *p* value
Age (years)				
≤55 y	18	1	100%	0.169
>55 y	14	1	88%	
FIGO substage				
IA	26	1	100%	0.019
IC	6	1	75%	
Surgery				
Radical surgery	25	1	95%	0.681
Fertility surgery	7	1	100%	
Tumor size				
<10 cm	23	1	100%	0.083
≥10 cm	7	1	83%	
Dominant growth pattern AGCT				
Diffuse	14	2	90%	0.183
Others	18	0	100%	
Grade of cytologic atypia				
1	15	0	100%	0.256
2 and 3	17	2	93%	
Mitotic index				
<4	17	1	100%	0.662
≥4	15	1	92%	
LVSI				
Negative	22	0	100%	0.022
Positive	10	2	83%	
Necrosis				
Absent	26	1	100%	0.040
Present	6	1	80%	
Hemorrhage				
Absent	23	0	100%	0.017
Present	9	2	83%	
Prominent fibrothecomatous component				
Absent	22	1	100%	0.530
Present	10	1	88%	

**Table 5:** Univariate analysis of disease-free survival in patients with early-stage adult granulosa cell tumors.

Features	*N*	Number of documented recurrence	% 5-year DFS	Log-rank test (*χ*^2^-test) *p* value
Age (years)				
≤55 y	18	2	94%	0.624
>55 y	14	2	83.5%	
FIGO substage				
IA	26	1	100%	0.0002
IC	6	3	42%	
Surgery				
Radical surgery	25	1	95%	0.004
Fertility surgery	7	3	71%	
Tumor size				
<10 cm	23	3	90.5%	0.860
≥10 cm	7	1	83.5%	
Dominant growth pattern AGCT				
Diffuse	14	4	76%	0.012
Others	18	0	100%	
Grade of cytologic atypia				
1	15	0	100%	0.032
2 and 3	17	4	79.5%	
Mitotic index				
<4	17	2	94%	0.736
≥4	15	2	83.5%	
LVSI				
Negative	22	1	95%	0.058
Positive	10	3	78%	
Necrosis				
Absent	26	3	92%	0.609
Present	6	1	80%	
Hemorrhage				
Absent	23	1	95%	0.022
Present	9	3	76%	
Prominent fibrothecomatous component				
Absent	22	2	96%	0.396
Present	10	2	77%	

## Data Availability

All the data necessary to verify the results of the study “Adult Granulosa Cell Tumors of the Ovary: a Retrospective Study of 36 FIGO Stage I Cases with Emphasis on Prognostic Pathohistological Features” are included within the article, and also all of them are available from the corresponding author upon request.
